# What the papers say

**DOI:** 10.1093/jhps/hnac009

**Published:** 2022-03-16

**Authors:** Ali Bajwa

**Affiliations:** Consultant Orthopaedic Surgeon, Villar Bajwa Practice, The Princess Grace Hospital, London, UK

The *Journal of Hip Preservation Surgery* (*JHPS*) is not the only place where work in the field of hip preservation can be published. Although our aim is to offer the best of the best, we are continually fascinated by work, which finds its way into journals other than our own. There is much to learn from it, and so *JHPS* has selected six recent and topical subjects for those who seek a summary of what is taking place in our ever-fascinating world of hip preservation. What you see here are the mildly edited abstracts of the original articles, to give them what *JHPS* hopes is a more readable feel. If you are pushed for time, what follows should take you no more than 10 min to read. So here goes …

## Safety and Efficacy Of A Single Intra-Articular Injection Of Hyaluronic Acid In Osteoarthritis Of The Hip: A Case Series Of 87 Patients

The authors from Australia [1] report that the osteoarthritis (OA) is the most prevalent form of joint disease and commonly affects the hip. Hip OA is associated with a high socioeconomic burden. Intra-articular hyaluronic acid (HA) injection may be of benefit, but quality evidence for HA use in hip OA is lacking. The purpose of this study was to assess the safety and efficacy of ultrasound guided injection of a high-molecular-weight, non-animal derived, stabilized HA (NASHA) in patients with mild-to-moderate hip OA.

This single site study is an analysis of prospectively collected outcome data for 87 consecutive patients over a 2-year period who received a single HA (Durolane) injection for symptomatic hip OA. Inclusion criteria were male or female patients over 18 years of age with mild–to-moderate hip OA on x-ray. Patients with severe hip OA were excluded. The primary outcome measure was a modified Harris Hip Score (mHHS) questionnaire at baseline and 6 weeks with a minimal clinically important difference (MCID) of 10 points. All adverse events were recorded and assessed.

Data from 87 patients, 49 women and 38 men with mean age of 54 (SD = 10.8), were analysed. At baseline, mean mHHS was 58.47 (SD 14.31). At the 6-week follow-up, mean mHHS improved to 71.30 (SD 16.46), a difference of 12.83 (*P* < 0.01). This was greater than the MCID of 10. No significant adverse events were encountered. Five patients reported short-lived injection site pain.

The authors concluded that a single injection of HA (NASHA) in the setting of hip joint OA was both safe and efficacious in this 87 patient cohort. Improvement in pain and function as measured with mHHS was statistically significant and reached the MCID of 10.

## Muscle and Hip Contact Forces In Asymptomatic Men With Cam Morphology During Deep Squat

In this study, the authors from Ottawa, Canada [2], note that cam morphology is defined as an aspherical femoral head–neck junction that causes abnormal contact of the acetabular rim with the anterior hip. Imaging confirmation of the cam morphology, associated with clinical signs and pain in the hip or groin, is characterized as femoroacetabular impingement syndrome (FAIS). Although some individuals with cam morphology do not experience any symptoms, sparse studies have been done on these individuals. Understanding the way asymptomatic individuals generate muscle forces may help us to better explain the progression of the degenerative FAI process and discover better ways in preventing the onset or worsening of symptoms. The purpose of this study was to compare the muscle and hip contact forces of asymptomatic cam morphology (ACM) and FAIS men compared to cam-free healthy controls during a deep squat task.

This prospective study compared 39 participants, with 13 in each group (ACM, FAI and control). Five deep squatting trials were performed at a self-selected pace while joint trajectories and ground reaction forces were recorded. A generic model was scaled for each participant, and inverse kinematics and inverse dynamics calculated joint angles and moments, respectively. Muscle and hip contact forces were estimated using static optimization. All variables were time normalized in percentage by the total squat cycle, and both muscle forces and hip contact forces were normalized by body weight. Statistical non-parametric mapping analyses were used to compare the groups.

The ACM group showed increased pelvic tilt and hip flexion angles compared to the FAI group during the descent and ascent phases of the squat cycle. Muscle forces were greater in the ACM and control groups, compared to the FAI group for the psoas and semimembranosus muscles. Biceps femoris muscle force was lower in the ACM group compared to the FAI group. The FAI group had lower posterior hip contact force compared to both the control and ACM groups. Muscle contraction strategy was different in the FAI group compared to the ACM and control groups, which caused different muscle force applications during hip extension.

The authors concluded that these results rebut the concept that mobility restrictions are solely caused by the presence of the cam morphology and propose evidence that symptoms and muscle contraction strategy can be the origin of the mobility restriction in male patients with FAI.

## Association Between Comorbid Depression and Rates Of Postoperative Complications, Readmissions and Revision Arthroscopic Procedures After Elective Hip Arthroscopy

Freshman *et al.* [3] report from USA that the depression and related psychiatric diagnoses are common in patients undergoing hip arthroscopy (HA) for FAIS. The effects of depression on postoperative complications, readmissions and additional ipsilateral hip surgery are not well studied.

Their hypothesis was that the patients with preoperative depression who undergo HA for FAIS would experience higher rates of 90-day postoperative complications and readmissions, with an increased risk of additional ipsilateral hip procedures, as compared with patients without depression.

They performed a retrospective cohort study between 2010 and 2019 using the Mariner/PearlDiver database. Current Procedural Terminology and International Classification of Diseases codes were used to compare patients with and without pre-existing depression who underwent HA for FAIS. Patients were matched at a 1:1 ratio based on age, sex, Charlson Comorbidity Index, body mass index and tobacco use. Patients undergoing shoulder or knee arthroscopy were also identified to compare lifetime preoperative depression prevalence amongst groups.

The lifetime preoperative depression prevalence was significantly higher in patients undergoing HA as compared with patients undergoing shoulder or knee arthroscopy (25.4% versus 22.2% versus 19.8%). When compared with the patients without depression, patients with preoperative depression had higher rates of 90-day readmissions (2.4% versus 1.5%) and complications, including urinary tract infection (36.2% versus 28.9%), pneumonia (12.9% versus 9.1%), hematoma formation (3.1% versus 1.9%), acute kidney injury (4.0% versus 2.6%), deep venous thrombosis/pulmonary embolism (2.6% versus 1.7%) and superficial infection (4.9% versus 2.8%), which were all statistically significant. Preoperative depression was associated with significantly higher odds of undergoing revision HA within 2 years (6.3% versus 2.4%).

The authors thus concluded that the patients with pre-existing depression experienced higher rates of 90-day postoperative complications and hospital readmissions after elective HA for FAIS and were more likely to undergo revision HA within 2 years of the index procedure.

## Effect Of Baseline Mental Health On 1-Year Outcomes After Ha: A Prospective Cohort Study

In this study, Lynch *et al.* [4] report that the patient factors, including mental health, sex and smoking, have been found to be more predictive of preoperative hip pain and function than intra-articular findings during HA for FAI. However, little is known about how these factors may influence patients’ postoperative outcomes.

They hypothesized that lower patient-reported mental health scores would be significant risk factors for worse patient-reported outcomes (PROs) 1 year after arthroscopic hip surgery for FAI and that baseline intra-articular pathology would fail to demonstrate an association with outcomes 1 year after FAI surgery.

They electronically enrolled a prospective cohort of patients undergoing HA for FAI. Baseline and 1-year follow-up PROs were collected, including Hip disability and Osteoarthritis Outcome Score for pain (HOOS-Pain), HOOS-Physical Function Short Form (HOOS-PS) and Veterans RAND 12-Item Health Survey–Mental Component Score (VR-12 MCS). Intra-articular operative findings and treatment were documented at the time of surgery. Proportional odds logistic regression models were built for 1-year outcomes (HOOS-Pain, HOOS-PS and VR-12 MCS). Risk factors included patient characteristics and intraoperative anatomic and pathologic findings.

Overall, 494 patients underwent HA for FAI, and 385 (78%) were evaluated at 1 year with at least 1 PRO. The median patient age was 33 years, mean body mass index was 25.5 kg/m^2^ and 72% were female. Multivariable analysis demonstrated that better baseline HOOS-Pain, HOOS-PS and VR-12 MCS were significantly associated with improvement in the 1-year scores for each PRO. Higher VR-12 MCS was significantly associated with better 1-year HOOS-Pain and HOOS-PS, while current and former smokers had worse 1-year outcomes than those who never smoked. In ranking each variable’s relative importance, baseline HOOS-Pain and HOOS-PS and baseline VR-12 MCS were identified as the strongest predictors of 1-year HOOS-Pain and HOOS-PS in our multivariable model.

The authors concluded that during HA for FAI, patient factors, including baseline hip pain and function, mental health and smoking, were independently associated with 1-year PROs of hip pain and function, while intra-articular pathology such as the presence of labral tear and its treatment, tear size, tear location and anchors placed were not independently associated.

## Resident Involvement In Ha Procedures Does Not Affect Short-Term Surgical Outcomes

The authors from New York, USA [5], evaluate whether the presence of residents in HA procedures affects short-term surgical outcomes.

The American College of Surgeons National Surgical Quality Improvement Program Database was used to identify patients who underwent HA from 2006 to 2012. Demographic and 30-day outcome variables were compared between cohorts of patients with and without residents. Multivariate logistic regression was used to identify whether resident involvement was an independent risk factor for adverse outcomes. Propensity score matching was performed to control for all demographic and intraoperative variables.

A total of 869 patients (59.7% female) were included in this study, 626 of which reported data on resident involvement. Patients were mostly White (51.8% with a resident). Those with residents were younger, had lower modified 5-item frailty index scores and had fewer cardiac comorbidities. There was no difference in diabetic status, dyspnoea symptoms, history of chronic obstructive pulmonary disease, renal comorbidity, neurologic comorbidity, cumulative comorbidities, history of bleeding disorders, inpatient versus outpatient treatment, preoperative functional status, smoking history and steroid use for chronic conditions. There was no difference in all complications, operative time, length of stay, reoperation, readmission, wound complication, venous thromboembolism, blood transfusions or sepsis. Propensity score match for demographic and intraoperative differences found no association between resident involvement and increased complications. Resident involvement was not an independent risk factor for all complications studied.

On the basis of their findings, the authors concluded that the resident involvement in HA procedures was not a risk factor for 30-day complications between 2006 and 2012. Resident involvement did not increase the risk of adverse outcomes, readmission, reoperation or length of stay, nor did it significantly increase operative times.

## Repair Versus Debridement For Acetabular Labral Tears—a Systematic Review

The authors Long *et al.* [6] state that the purpose of this study was to systematically review the evidence in the literature to ascertain whether acetabular labral repair (ALR) or debridement (ALD) resulted in superior patient outcomes.

The systematic review was conducted in accordance with the Preferred Reporting Items for Systematic Reviews and Meta-Analyses guidelines. Peer-reviewed studies comparing ALR and ALD published in English with full text available were included. Patients undergoing both open and arthroscopic surgery in randomized controlled trials, prospective cohort studies, retrospective cohort studies and case–control studies were included. Studies were quantified for methodological quality using the MINORS criteria. Clinical outcomes were compared, with qualitative analysis, and quantitative analyses were performed using GraphPad Prism version 7. A *P* value <.05 was considered to be statistically significant.

They included 8 studies (level of evidence [LOE] I = 1; LOE II = 2; LOE III = 5). The 7 studies compared 364 patients (369 hips) with ALR to 318 patients (329 hips) with ALD, with a mean follow-up time ranging between 32 and 120 months. Five studies found significantly improved PROs with ALR (Harris Hip Score, Merle d’Aubigné, Pain, SF-12). Several studies compared the outcomes after ALR and ALD and found statistical significance in all investigated metrics in favour of ALR. One study found a significant improvement in abduction, but no other study found any difference in range of motion. No study found any difference in complication rate, revision rate or conversion to total hip arthroplasty. However, two studies found ALR reduced the rate of osteoarthritic progression.

The authors thus concluded that the current literature suggests that ALR may result in superior PROs. However, there appears to be no significant difference in the rate of progression to total hip arthroplasty at up to 10-year follow-up.
